# The Modified Glasgow Prognostic Score Predicts Survival in Gastric Cancer Patients with Normal CEA and CA19-9

**DOI:** 10.1155/2022/3953004

**Published:** 2022-06-13

**Authors:** Shun Zhang, Jing-Ze Li, Tao Du, Hai-Qiang Li, Ren-Hao Hu, Chi-Ye Ma, Xi-Mao Cui, Chun Song, Xiao-Hua Jiang

**Affiliations:** ^1^Department of Gastrointestinal Surgery, Shanghai East Hospital, Tongji University, Shanghai, China; ^2^Center of Digestive Endoscopy, Shanghai East Hospital, Tongji University, Shanghai, China

## Abstract

**Background:**

Traditionally, serum CEA and CA19-9 levels are good prognostic factors for gastric cancer. Many gastric cancer patients do not have elevated CEA or CA19-9 levels even at a very advanced stage. This study investigates the significance of the modified Glasgow prognostic score (mGPS) for the survival of gastric cancer patients with normal CEA and CA19-9.

**Methods:**

We retrospectively examined 488 curatively resected gastric cancer patients with normal preoperative serum levels of CEA and CA19-9 to evaluate the prognostic ability of mGPS for overall survival. The prognostic significance was analyzed by univariate and multivariate analyses.

**Results:**

Age, hemoglobin, white cell count, and neutrophils were each significantly correlated with the mGPS. Multivariate analyses showed that tumor location (HR, 0.803; 95% CI, 0.667–0.966; *P*=0.020), TNM stage (HR, 2.714; 95% CI, 2.250–3.275; *P* < 0.001), and mGPS (HR, 1.042; 95% CI, 1.105–1.772; *P*=0.023) were significantly associated with overall survival. Significant correlations were found between overall survival and mGPS. The Kaplan–Meier analysis demonstrated significant differences among patients with mGPS of 0, 1, and 2 (*P* < 0.001), with the mortality rate being higher for patients with a higher mGPS.

**Conclusion:**

The mGPS can predict survival in gastric cancer patients with normal CEA and CA19-9.

## 1. Introduction

Gastric cancer (GC) is the fifth most frequently diagnosed malignancy worldwide, especially in Asia, even though the incidence and mortality of gastric cancer have fallen dramatically in the last 20 years [1]. Despite the advances in diagnostic methods and the improvement of surgical treatments, systemic chemotherapy, targeted treatments, and immunotherapies, the prognosis remains unsatisfactory, with a reported 5-year overall survival (OS) rate after resection of less than 70% [2]. Identifying reliable and powerful biomarkers to predict recurrence and poor prognosis may improve treatment efficiency and patients' outcomes.

TNM staging is no doubt the most established prognostic factor for GC. However, it provides limited information on the disease progression of individual patients [3]. Multiple serum tumor markers, such as carcinoembryonic antigen (CEA) and CA19-9, are commonly used in clinical practice to predict the prognosis and survival of GC patients [4–6]. However, even patients with advanced stages may not have increased serum levels of CEA or CA19-9 in the perioperative period. Therefore, these serum tumor markers cannot be widely applied for screening tumor status and prediction of postoperative survival in certain group of patients. Recent findings have demonstrated the superiority and utility of micro-RNAs (miRNAs) as new biomarkers for cancer diagnosis, therapy, and prognosis [7]. Among the known miRNAs, miR-135a with the upregulation of VEGF signaling has been indicated as a tumor suppressor in gastric cancer, whereas its role as biomarker in GC is under investigation [7, 8]. In recent years, growing evidence has shown that systemic inflammation plays an important role in various types of tumor development and progression. In particular, it has been shown that the modified Glasgow prognostic score (mGPS) can be a useful biomarker of survival for GC patients after surgery [9].

To our knowledge, few studies reported the utility of mGPS in other types of cancer with normal preoperative serum like CEA and CA19-9, and none investigated the potential predictive performance of mGPS for GC in this setting. Therefore, we retrospectively analyzed 488 GC patients who received gastrectomy and evaluated the prognostic ability of preoperative mGPS in patients with normal serum CEA and CA19-9 levels.

## 2. Materials and Methods

### 2.1. Patients

Patients with histologically confirmed gastric cancers who had undergone gastrectomy at the Department of Gastrointestinal Surgery of Shanghai East Hospital between August 2006 and December 2016 were retrospectively reviewed. All patients who had undergone gastrectomy and routine preoperative hematological testing of C-reactive protein (CRP), albumin (ALB), CEA, and CA19-9 were available and further analyzed. Finally, patients with preoperative serum levels of CEA ≤5.0 ng/ml and CA19-9 ≤30 U/ml were enrolled in the present study. The clinicopathological classification of gastric cancer was according to the 8th edition of the American Joint Committee on cancer tumor-node-metastasis (TNM) classification [10]. The retrospective protocol of this study was approved by the Ethical Review Board of Shanghai East Hospital. Informed consent from patients was waived due to the retrospective nature of our cohort study.

### 2.2. mGPS Evaluation

The mGPS was calculated using CRP and ALB levels as previously described. In brief, patients with both an elevated CRP (>10 mg/L) and hypoalbuminaemia (<35 g/L) were allocated a prognostic score of 2, patients with an elevated CRP alone were assigned mGPS 1, and patients with a normal CRP regardless of the ALB levels were assigned mGPS 0.

### 2.3. Patient Follow-Up

All patients were regularly followed up with medical history, physical examination, computed tomography, and laboratory testing every 3 months at the first 2 years postoperatively and then every 6 months for more than 2 years.

### 2.4. Statistical Analysis

Descriptive statistics were performed using mean, 95% confidence intervals (CIs), and percentages. Univariate overall survival (OS) analysis was first calculated using the Kaplan–Meier method and log-rank test. In order to exclude other confounding factors affecting survival, the prognostic characters of *P* value <0.05 identified by univariate analysis were further confirmed by multivariate analysis. Multivariate survival analysis was performed using the Cox proportional hazard ratio (HR) model. Statistical analysis was performed using SPSS Statistics 18.0 software (IBM SPSS, Inc., Chicago, IL, USA) at a significance level of *P* value <0.05.

## 3. Results

### 3.1. Relationships between Clinicolaboratory Characteristics and mGPS

Because of the extremely low rate of higher scores of mGPS in stage Ia patients, we cannot perform the comparison of the mGPS value between different scores in those patients. Thus, we did not include stage Ia patients in our study. A total of 488 patients were finally enrolled in this study. Of the 488 patients, 337 (69.1%) were males and 151 (30.9%) were females. The primary tumors were located in the upper third of 158 (32.4%) patients, the middle third of 192 (39.3%) patients, and the lower third of 138 (28.3%) patients. And, the tumor was classified as stage Ib in 69 (14.1%) patients, stage II in 139 (28.5%) patients, stage III in 161 (33.0%) patients, and stage IV in 119 (24.4%) patients, respectively. There were no significant differences between groups mGPS0, mGPS1, and mGPS2 in terms of most patient characteristics, with the exceptions of age (*P*=0.044), hemoglobin (*P* < 0.001), white cell count (*P* < 0.001), and neutrophils (*P* < 0.001) (Table 1).

### 3.2. Clinical and Laboratory Characteristics Associated with OS

The characteristics and OS analysis of those patients are shown in Table 2. Tumor location at the middle third had the longest survival time, with 82.7 months, followed by lower third tumors (78.1 months) and upper third tumors (60.5 months). The mean survival for stage Ib, stage II, stage III, and stage IV was 103.9, 83.6, 72.4, and 31.7 months, respectively. The mean survival of patients with an mGPS of 0, 1, and 2 was 76.4, 64.9, and 37.9 months, respectively. There was no significant difference in OS in terms of sex, age, BMI, white cell count, neutrophils, and lymphocytes. It was shown that OS had a significant correlation with tumor location (*P*=0.001), hemoglobin (*P*=0.001), TNM stages (*P* < 0.001), and mGPS (*P*=0.001).

### 3.3. Prognostic Value of the Clinicolaboratory Characteristics and mGPS for OS

As shown in Table 3, univariate analyses indicated that the following clinical characteristics were significant prognostic factors for OS in GC patients: tumor location (HR = 0.806; 95% CI = 0.667–0.974; *P*=0.026), hemoglobin (HR = 0.626; 95% CI = 0.471–0.832; *P*=0.001), tumor stage (HR = 2.736; 95% CI = 2.279–3.285; *P* < 0.001), and mGPS (HR = 1.445; 95% CI = 1.117–1.869; *P*=0.005). Further multivariate analysis based on the previously mentioned parameters confirmed that tumor location (HR = 0.803; 95% CI = 0.667–0.966; *P*=0.020), tumor stage (HR = 2.714; 95% CI = 2.250–3.275; *P* < 0.001), and mGPS (HR = 1.042; 95% CI = 1.105–1.772; *P*=0.023) were independent predictors of OS.

### 3.4. OS Analysis Stratified by mGPS

**Figure 1:**
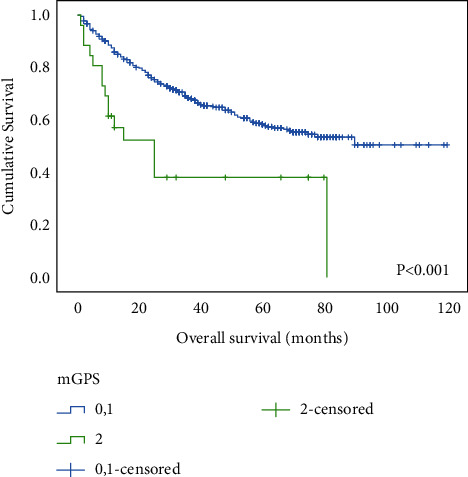
Relationship between the mGPS and overall survival in patients with gastric cancer.

**Figure 2:**
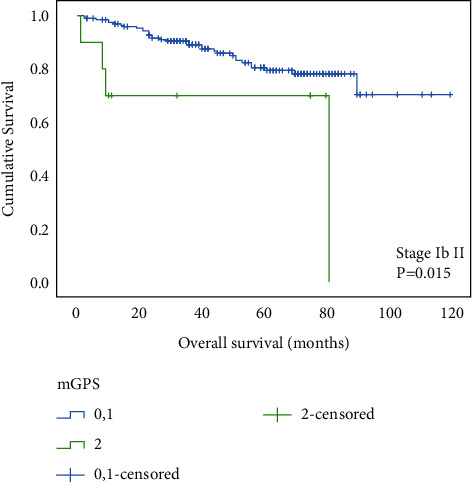
Relationship between the mGPS and overall survival in patients with stage Ia and II gastric cancer.

**Figure 3:**
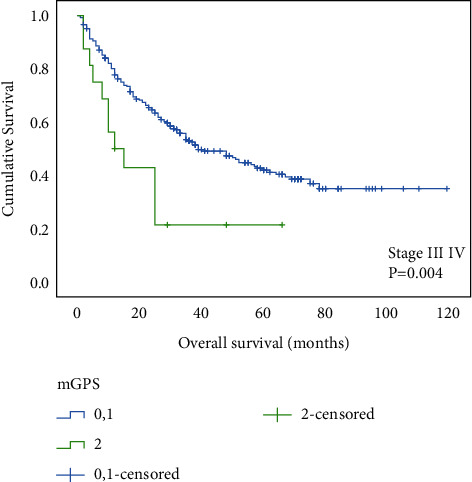
Relationship between the mGPS and overall survival in patients with stage III and IV gastric cancer.

Our results indicate that patients with an mGPS of 2 had significantly shorter OS than those with an mGPS of 0 or 1 (*P* < 0.001) (Figure 1). To evaluate the prognostic value of mGPS depending on tumor stage, all patients were then stratified into two groups, namely, those with relatively early-stage tumors (stage Ib and II) and those with advanced-stage tumors (stage III and IV). We found that mGPS 2 demonstrated significantly shorter OS not only in patients with stage Ib plus II but also with III combined with IV (Figures 2 and 3).

## 4. Discussion

There is continued interest in identifying simple, feasible, and low-cost markers to permit more accurate patient stratification, which will allow for improved clinical decision-making. The present retrospective study analyzed whether mGPS measured before surgery can be used as a prediction factor for postoperative survival in GC patients with normal serum CEA and CA19-9 levels. Of interest, univariate analysis and further multivariate analyses revealed that mGPS was significantly associated with OS, indicating that mGPS may be a useful predictor of postoperative outcome for GC.

The AJCC TNM classification is the most commonly used staging method for the prognosis of gastric cancer. However, it is still difficult to obtain complete prognostic information [11]. For many years, some studies have reported that preoperative elevated CEA or/and CA19-9 are related to GC metastasis and prognosis [12, 13]. CEA is a glycoprotein attached to the surface of enterocytes with a role in programmed cell death and cell adhesion [14]. CEA is used predominantly for the management of colorectal carcinoma, and its levels may be increased in gastric, lung, pancreatic, and breast carcinomas. High pretherapeutic levels of CEA are correlated with the stage of the disease. Furthermore, CEA is of diagnostic power in postoperative follow-up and early detection of recurrent disease. Although CEA is an important tumor marker in cancer, its serum levels can also be elevated in benign diseases [15]. CA 19-9, also known as sialyl Lewis antigen, is synthesized in the normal pancreatic parenchyma and biliary tract. It is also produced by the epithelial cells of the gastric, colonic, and uterine mucosa, as well as the salivary glands [16]. CA19-9 increases to the greatest extent in patients with pancreatic and biliary tract tumors [17]. Among digestive tract cancers, elevated serum CA19-9 was observed in a relatively varying proportion (7.3–18%) of patients with GC [18, 19], and elevated CA 19-9 levels have been significantly correlated with lymph node metastasis, vascular invasion, and liver metastasis [16]. Additionally, it may also be increased in several benign diseases, including pancreatitis and cholelithiasis, pulmonary and thyroidal diseases, diabetes mellitus, and gynecologic diseases [20]. The tumor markers CEA and CA19-9 are widely used for real-world opportunistic cancer screening. However, the sensitivity of these markers is reportedly low considering single-organ cancer [21]. Both markers are not recommended by any guidelines for cancer screening, especially due to their low sensitivity for early gastric cancer [21]. The positive rates of both markers were unsatisfactory, especially in early gastric cancer. It was reported that the positive rate was 4.3% to 14.5% for CEA [12, 22, 23], and 4.8% to 12.3% for CA19-9 [12, 22, 23] for early gastric cancer. In advanced gastric cancer, the positive rates of the serum tumor markers varied widely. The positive rates of CEA ranged from 34.5% to 47.8% [22–24] and the CA19-9 level was 29.8% to 40.2% [22–24]. Moreover, many patients with metastatic GC may not have elevated CEA or/and CA19-9 perioperation. Therefore, the unsatisfactory specificity and sensitivity of CEA or/and CA19-9 may limit clinical utility. It is warranted to find novel biomarkers for these patients.

The mGPS, a cumulative prognostic score based on the combination of serum CRP and ALB levels, might reflect both systemic inflammatory response and physical nutritional decline [9]. The association between cancer and systemic inflammation has been widely explored for decades, and accumulating studies have elucidated that the presence of a systemic inflammatory response is a useful predictor of poor outcome in various malignancies [25]. Additionally, serum ALB level as a leading marker for nutritional status may be influenced by a systemic inflammatory response [26]. Accumulating studies have elucidated mGPS can be a potent and reliable prognostic biomarker in GC. Recent studies show that mGPS may serve as a valuable prognostic factor of survival, independent of TNM stage for patients with esophageal cancer [27] and colorectal cancer [28] with a normal preoperative CEA level. Accumulating studies have elucidated that GPS can be a potent and reliable prognostic biomarker in GC and as a predictive factor for adjuvant chemotherapy in gastric cancer patients after curative surgery [29], for which the immune response has been implicated as a key determinant. GPS has been reported to correlate with elevated cytokine levels, adipokine levels, drug metabolism, weight and muscle loss, and poor performance status [30]. These factors may be related to the immune status of the host, and they may affect the efficacy of antitumor immunity therapy. Emerging evidence indicates that angiogenesis and immunosuppression frequently occur simultaneously during tumor growth and evolution through constant crosstalk with the surrounding microenvironment [31]. Recent study suggests that mGPS may serve as a biomarker to reflect sensitivity to immune checkpoint inhibitors in advanced GC and renal cancer [30, 32]. However, no other study has explored whether mGPS can be used for predicting postoperative survival in GC patients with normal CEA and CA19-9.

Stage Ia patients were not included in our study due to the extreme higher rate of mGPS 0. Stage Ib, II, III, and IV accounted for 14.9%, 28.1%, 32.7%, and 24.3% of the 523 enrolled patients, respectively. In the present study, 10.3% of patients were assigned an mGPS of 1 or 2. The mean survival time of patients with an elevated mGPS score was significantly lower than that of patients with a lower mGPS score, which emphasizes the correlation between mGPS and prognosis. With regard to follow-up, patients with a normal CEA and CA19-9 were further stratified into two groups: those with stage Ib or II and those with stage III or IV. Our results indicated that higher mGPS (1 or 2) was related to poor survival in GC patients with advanced stage (stage III or IV). Similar to the results of patients with stage III or IV gastric cancer, our results also indicated significant postoperative survival differences depending on the mGPS in stage Ib or II patients. This finding implies that the mGPS might also have prognostic value for postoperative survival in GC patients with a relatively early stage.

There are also some limitations to our study. First, selection bias may be present because this was a single-institution, retrospective study. It is still necessary to perform multi-institutional prospective studies to assess the prognostic value of mGPS. Second, we only focused on the preoperative mGPS on postoperative survival without evaluating their preoperative to postoperative changes. Third, we only explore the value of mGPS in OS other than RFS. Recent studies indicate a survival discrepancy exists between first relapse-related events (DFS or PFS) and OS in cancer patients who received treatment [33]. Fourth, other biomarkers associated with inflammation and nutrition were not analyzed. It remains to be established whether nutritional support can improve the prognoses of GC patients.

## 5. Conclusion

In conclusion, the results of our study suggest that the mGPS could be a simple and useful predictor in GC patients and can be a significant prognostic factor in the estimation of tumor characteristics in patients with normal CEA and CA19-9.

## Figures and Tables

**Table 1:** Relationships between clinicolaboratory characteristics and the modified Glasgow prognostic score (mGPS).

	mGPS = 0 *n* (%)	mGPS = 1 *n* (%)	mGPS = 2 *n* (%)	*P* value^†^
Sex				
Male	297 (88.1)	19 (5.7)	21 (6.2)	0.132
Female	142 (94.0)	4 (2.6)	5 (3.4)	

Age (years)				
<65	239 (93)	7 (2.7)	11 (4.3)	0.044
≥65	200 (86.6)	16 (6.9)	15 (6.5)	

Body mass index (kg (m^2^)^−1^)				
<25	351 (88.9)	19 (4.8)	25 (6.3)	0.057
≥25	88 (94.6)	4 (4.3)	1 (1.1)	

Tumor location				
Upper third	139 (88)	7 (4.4)	12 (7.6)	0.578
Middle third	176 (91.7)	8 (4.2)	8 (4.2)	
Lower third	124 (89.9)	8 (5.8)	6 (4.3)	

Hemoglobin (g/L)				
<12	174 (82.1)	16 (7.5)	22 (10.4)	<0.001
≥12	265 (96.0)	7 (2.5)	4 (1.4)	

White cell count (×10^9^ L^−1^)				
<11	436 (91.6)	19 (4.0)	21 (4.4)	<0.001
≥11	3 (25.0)	4 (33.3)	5 (41.7)	

Neutrophils (×10^9^ L^−1^)				
<7.5	431 (91.7)	21 (4.5)	18 (3.8)	<0.001
≥7.5	8 (44.4)	2 (11.1)	8 (44.4)	

Lymphocytes (×10^9^ L^−1^)				
<3	420 (90.1)	21 (4.5)	25 (5.4)	0.668
≥3	19 (86.4)	2 (9.1)	1 (4.5)	

TNM				
Ib	65 (94.2)	3 (4.3)	1 (1.4)	0.357
II	124 (89.2)	6 (4.3)	9 (6.5)	
III	148 (91.9)	7 (4.3)	6 (3.7)	
IV	102 (85.7)	7 (5.9)	10 (8.4)	

^†^ *χ* ^2^ test.

**Table 2:** Clinical and laboratory characteristics associated with overall survival.

	No. of patients	Overall survival (months) Mean (95% CI)	*P* value^†^
Sex			
Male	337	75.3 (69.4–81.2)	0.685
Female	151	73.8 (65.1–82.6)	

Age (years)			
<65	257	70.3 (64.0–76.7)	0.573
≥65	231	76.8 (69.5–84.1)	

Body mass index (kg/m^2^)			
<25	395	74.0 (68.4–79.7)	0.689
≥25	93	68.9 (60.4–77.4)	

Tumor location			
Upper third	158	60.5 (52.5–68.4)	0.001
Middle third	192	82.7 (75.1–90.3)	
Lower third	138	78.1 (69.1–87.0)	

Hemoglobin (g/L)			
<12	212	66.3 (58.7–73.8)	0.001
≥12	276	80.8 (74.1–87.5)	

White cell count (×10^9^/L)			
<11	476	74.8 (69.7–79.9)	0.831
≥11	12	58.9 (41.6–76.2)	

Neutrophils (×10^9^/L)			
<75	470	75.0 (69.8–80.1)	0.570
≥7.5	18	62.8 (41.1–84.5)	

Lymphocytes (×10^9^/L)			
<3	466	74.3 (69.1–79.5)	0.255
≥3	22	85.3 (68.0–102.7)	

TNM			
Ib	69	103.9 (94.7–113.1)	<0.001
II	139	83.6 (77.0–90.2)	
III	161	72.4 (65.1–79.7)	
IV	119	31.7 (24.3–39.1)	

mGPS			
0	439	76.4 (71.1–81.6)	0.001
1	23	64.9 (50.0–79.8)	
2	26	37.9 (23.6–52.3)	

^†^Kaplan–Meier survival analysis.

**Table 3:** Univariate and multivariate analyses of overall survival.

	Univariate analysis		Multivariate analysis			
	*P* value	Odds ratio	95% CI	*P* value	Odds ratio	95% CI
Sex	0.687	1.065	0.783–1.449			
Age	0.575	0.922	0.693–1.226			
Body mass index	0.690	0.930	0.649–1.331			
Tumor location	0.026	0.806	0.667–0.974	0.020	0.803	0.667–0.966
Hemoglobin	0.001	0.626	0.471–0.832	0.964	0.993	0.734–1.344
White cell count	0.832	0.908	0.373–2.209			
Neutrophils	0.572	1.226	0.604–2.490			
Lymphocytes	0.261	0.649	0.305–1.381			
Tumor stage (I/II/III/IV)	<0.001	2.736	2.279–3.285	<0.001	2.714	2.250–3.275
mGPS (0, 1, and 2)	0.005	1.445	1.117–1.869	0.023	1.042	1.105–1.772

## Data Availability

The data used to support the findings of this study are available from the corresponding author upon request.
